# Recent Advancements in Bone Tissue Engineering: Integrating Smart Scaffold Technologies and Bio-Responsive Systems for Enhanced Regeneration

**DOI:** 10.3390/ijms25116012

**Published:** 2024-05-30

**Authors:** Kelly M. Percival, Vinod Paul, Ghaleb A. Husseini

**Affiliations:** 1 Department of Chemical and Biological Engineering, American University of Sharjah, Sharjah P.O. Box 26666, United Arab Emirates; kpercival@aus.edu (K.M.P.); b00068146@aus.edu (V.P.); 2 Materials Science and Engineering Program, College of Arts and Sciences, American University of Sharjah, Sharjah P.O. Box 26666, United Arab Emirates

**Keywords:** bone tissue engineering, biomaterials, scaffold fabrication, bio-responsive scaffolds, regenerative medicine

## Abstract

In exploring the challenges of bone repair and regeneration, this review evaluates the potential of bone tissue engineering (BTE) as a viable alternative to traditional methods, such as autografts and allografts. Key developments in biomaterials and scaffold fabrication techniques, such as additive manufacturing and cell and bioactive molecule-laden scaffolds, are discussed, along with the integration of bio-responsive scaffolds, which can respond to physical and chemical stimuli. These advancements collectively aim to mimic the natural microenvironment of bone, thereby enhancing osteogenesis and facilitating the formation of new tissue. Through a comprehensive combination of in vitro and in vivo studies, we scrutinize the biocompatibility, osteoinductivity, and osteoconductivity of these engineered scaffolds, as well as their interactions with critical cellular players in bone healing processes. Findings from scaffold fabrication techniques and bio-responsive scaffolds indicate that incorporating nanostructured materials and bioactive compounds is particularly effective in promoting the recruitment and differentiation of osteoprogenitor cells. The therapeutic potential of these advanced biomaterials in clinical settings is widely recognized and the paper advocates continued research into multi-responsive scaffold systems.

## 1. Introduction

Bone tissue engineering (BTE) has emerged as a vital interdisciplinary field that addresses significant challenges in regenerative medicine by developing scaffolds that support and enhance bone regeneration. This domain synthesizes principles from biology, materials science, and engineering to innovate beyond the limitations of conventional bone repair methods, such as autografts and allografts. These methods often fail to meet clinical needs due to donor site morbidity, limited availability, potential for disease transmission, and suboptimal integration with host bone tissue—particularly in cases of extensive bone loss or complex defects [1,2,3,4,5,6]. Hence, advancements in scaffold design are prompted by the complexity of bone physiology and the need for scaffolds that can adequately mimic biological functions.

Recent advancements have seen significant progress in scaffold designs that incorporate dynamic and bio-responsive features, reflecting a shift towards scaffolds capable of adapting to and mimicking the natural extracellular matrix (ECM) of bone. This adaptability is pivotal, as static properties—such as mechanical stability, porosity, biocompatibility, and degradation rate—while essential, are designed to remain relatively unchanged throughout the healing process. These properties are indispensable for the effective integration and functionality of scaffolds in therapeutic contexts and ensure that scaffolds can support and guide bone growth effectively under normal physiological conditions [7,8]. However, the persistent focus on enhancing these static characteristics highlights a critical area for ongoing research and development, suggesting that fully harnessing the potential of dynamic responsiveness in scaffolds might more effectively meet the complex requirements of the bone healing process.

This review critically examines the development of novel, smart scaffold systems that incorporate both bioactive materials and responsive elements capable of adjusting their properties in real time, alongside a review of recent studies that have demonstrated significant progress in this field. These innovations are specifically designed to facilitate initial bone tissue integration and dynamically adapt to the evolving healing environment, thus promoting optimal regeneration throughout the recovery process. The significant advancements in BTE that provide scaffolds that are both structurally supportive and functionally dynamic represent substantial progression towards addressing the current limitations in the field. 

Following the introductory exploration of bone anatomy, physiology, remodeling, and repair, this review is structured into several sections to provide a comprehensive understanding of the current landscape and future directions in BTE. Section 2 discusses criteria for the selection of scaffold biomaterials, as well as polymers, ceramics, metals, and composites. Section 3 explores technological advancements in scaffold fabrication, such as 3D printing and additive manufacturing techniques, as well as surface modification and the integration of cells and bioactive molecules within the scaffold matrices. Section 4 includes a detailed investigation of recent strategies in bio-responsive scaffolds, whereby each strategy is examined for its potential to respond to different physical and chemical stimuli. Finally, Section 5 synthesizes the current trends and advancements, proposing directions for future research and potential clinical applications.

### 1.1. Bone Anatomy and Physiology

A bone is a rigid, mineralized organ that forms the skeleton of vertebrates and fulfills critical mechanical and biological functions. The human body has 206 bones that offer structural support, protect internal organs, and enable mobility. They also play key roles in blood cell production (hematopoiesis), storing minerals, and executing essential endocrine functions [9]. Bones are primarily composed of an osteoid matrix enriched with hydroxyapatite (HAp) [Ca_10_(PO_4_)_6_(OH)_2_] crystals, which impart structural hardness and strength [10]. This matrix also contains organic collagen fibers that provide flexibility, along with essential components such as water for nutrient and waste transport, non-collagenous proteins, lipids, and specialized cells that maintain bone health and functionality.

**Figure 1:**
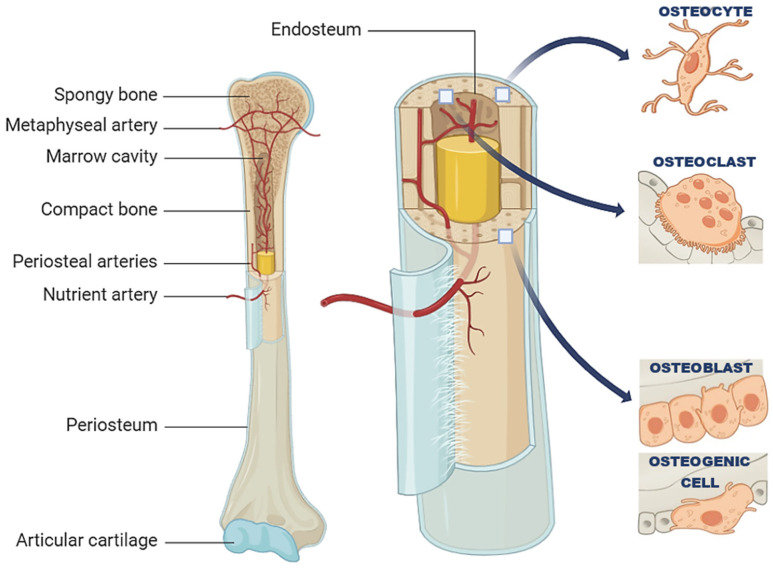
Structure of a typical long bone (created with biorender.com).

The process of bone development starts between the sixth and seventh weeks of embryonic growth through a process known as ossification or osteogenesis. Mesenchymal Stem Cells (MSCs), or mesenchymal progenitors, are multipotent stem cells found in the bone marrow and other tissues. MSCs have the capacity to differentiate into various cell types, including those of the osteogenic lineage. As shown in Figure 1, four essential cells play important roles in bone development and formation, each contributing distinctively to the bone’s lifecycle and functional integrity. Osteogenic cells, or osteoprogenitor cells, are the precursors in the osteoblastic lineage and reside primarily in the periosteum and endosteum—key sites for bone growth and repair. These cells differentiate into osteoblasts, which are responsible for bone formation. Osteoblasts synthesize and secrete an amorphous material that gradually solidifies to form osteoid, a form of unmineralized bone tissue, which is later mineralized through the deposition of calcium phosphate crystals in the formed osteoid matrix. Osteoblasts are essential in the mineralization process that solidifies the bone structure. As osteoblasts finish producing the bone matrix, they become embedded within it and transform into osteocytes [11]. Osteocytes are the most abundant cells in mature bone and function as mechanosensors and regulators of bone metabolism, maintaining the mineral content of the matrix. Finally, osteoclasts are multinucleated cells derived from the monocyte/macrophage lineage, responsible for bone resorption. Their activity is critical in bone remodeling, an ongoing process that balances bone formation (by osteoblasts) and resorption (by osteoclasts). This dynamic cycle continuously replaces old bone tissues with new ones and ensures the maintenance and renewal of the skeletal system throughout life.

### 1.2. Bone Remodeling

**Figure 2:**
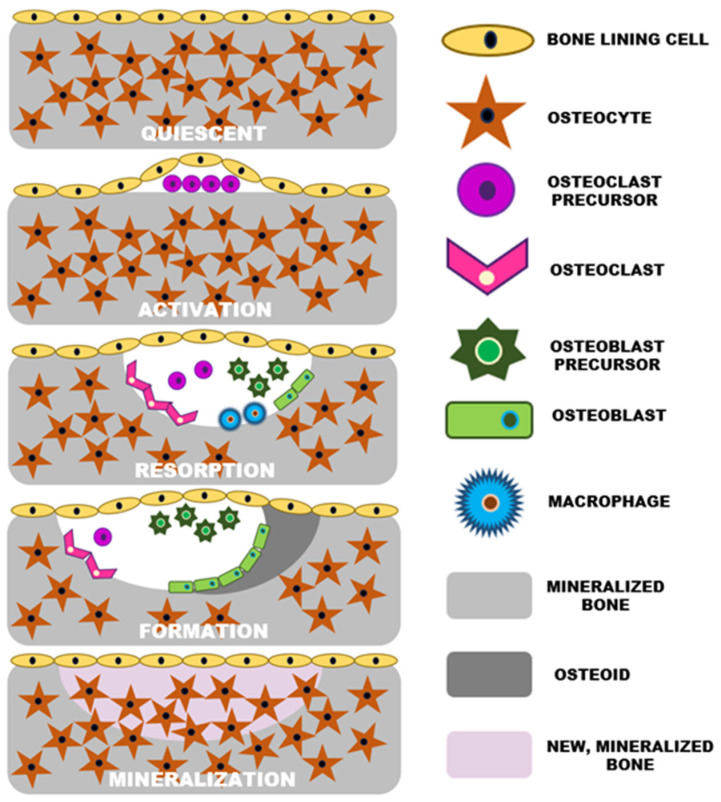
Stages of bone remodeling.

The bone is metabolically active tissue and undergoes a cyclic, continuous modeling process that is necessary for maintaining skeletal integrity and metabolic functions within vertebrates. As illustrated in Figure 2, the bone remodeling process is regulated by a series of five stages: activation, resorption, reversal, formation, and termination. Each is characterized by specific cellular activities and biochemical interactions.

The cycle initiates with the activation phase, where bone lining cells separate from underlying bone to form a raised canopy over the site to be resorbed [10]. Osteoclast precursors are recruited from circulation and activated at specific sites on the bone surface. This process is often stimulated by signals following the apoptosis of osteocytes, which are primary mechanosensing cells of bone that can initiate remodeling in response to microdamage or mechanical strain [12]. The osteoclast precursor cells then differentiate into multinucleated osteoclasts that firmly attach to the bone matrix, creating isolated resorption pits. During the resorption phase, osteoclasts secrete protons and enzymes, like cathepsin K, to dissolve bone minerals and degrade the matrix [10,13]. The programmed cell death of osteoclasts terminates at this stage to ensure that excess resorption does not occur. The reversal phase serves as a transition from bone resorption to formation. It involves the preparation of the bone surface for new bone deposition, primarily facilitated by cells of the osteoblastic lineage. These cells clean up the resorbed area, preparing it for new matrix deposition. The onset of the formation phase is marked by the synthesis of a new bone matrix (or osteoid) by osteoblasts. This osteoid, primarily composed of type I collagen, is subsequently mineralized through the deposition of HAp crystals. The regulation of this process is complex, involving systemic and local factors that control the availability of calcium and phosphate [10]. The completion of new bone formation leads to the differentiation of osteoblasts into bone-lining cells or their entrapment within the matrix as osteocytes. These newly formed osteocytes then secrete factors that signal the termination of the remodeling cycle. All aforementioned stages are crucial. Any abnormalities of bone remodeling can produce a variety of skeletal disorders such as osteoporosis, hyperparathyroidism, hyperthyroidism, Paget’s disease, osteopetrosis, and orthopedic disorders.

### 1.3. Bone Repair

**Figure 3:**
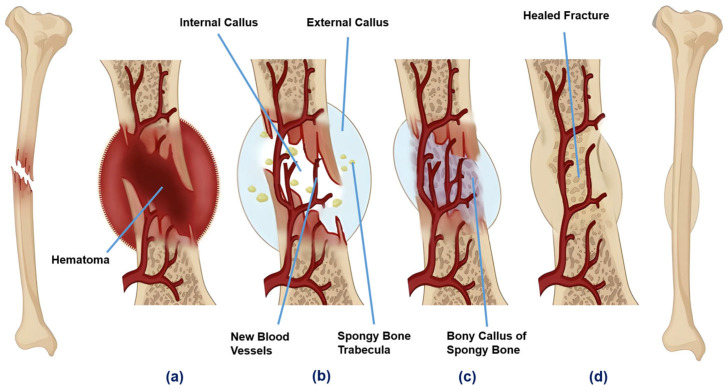
Four stages of fracture repair: (**a**) hematoma formation, (**b**) fibrocartilaginous callus formation, (**c**) bony callus formation, and (**d**) bone remodeling (created with biorender.com).

Depending on the nature and extent of the fracture, bone healing can be primary or secondary. Primary healing occurs when the fracture gap is minimal (less than 0.1 mm) and the fracture site is rigidly stabilized, allowing direct ossification without visible callus formation [14]. This type of healing is less common and typically requires surgical intervention to ensure the precise alignment and stabilization of the fracture. Secondary healing occurs when the fracture gap is wider but not exceeding twice the diameter of the bone [14,15]. This process involves a well-orchestrated series of biological events, leading to the formation of a callus, which subsequently matures to restore the bone’s structural integrity. While the underlying mechanisms of bone healing are complex, it is widely acknowledged that for successful healing, there must be viable osteogenic cells, an appropriate connective tissue matrix, sufficient vascularity, growth factors, and an adequate degree of mechanical support. As shown in Figure 3, the mechanism of fracture repair can be divided into four stages.

1. Hematoma formation (1–5 days)

After a fracture, ruptured blood vessels bleed into and around the fracture site, forming a hematoma. This clot seals the damaged blood vessels and forms a temporary frame for healing. The hematoma environment stimulates the release of pro-inflammatory cytokines, which attract immune cells like macrophages, monocytes, and lymphocytes. Such immune cells not only clear debris (dead cells) but also secrete vascular endothelial growth factor (VEGF). VEGF is a key mediator in stimulating angiogenesis (the formation of new blood vessels), necessary for subsequent healing stages. It specifically stimulates endothelial cells, which line the interior surface of blood vessels, to divide and migrate, leading to the growth of new capillary networks within the damaged tissue area.

2. Fibrocartilaginous callus formation (5–11 days)

Driven by the angiogenic cues from VEGF, new capillaries grow into the hematoma. This vascular invasion supports the influx of reparative cells, including fibroblasts and osteoblasts. Fibroblasts produce collagen fibers that span between the broken bone ends, while osteoblasts form spongy bone. The repair tissue between the broken bone ends is a semi-rigid structure known as the fibrocartilaginous callus. It is made up of hyaline and fibrocartilage and provides temporary stabilization of the fracture.

3. Bony callus formation (11–28 days)

The fibrocartilaginous callus is gradually resorbed into a bony callus of spongy bone, and the broken bone ends are firmly joined together. This process is similar to the formation of bone from cartilage and involves the coordinated activity of osteoblasts, which continue to deposit new bone, and osteoclasts and remodel the immature bone to enhance structural integrity. At the end of this stage, a hard, immature callus bone forms.

4. Bone remodeling (18 days to years)

The final phase involves the remodeling of the bony callus into mature lamellar bone, restoring the bone’s natural architecture. This prolonged phase ensures the newly formed bone can withstand normal physiological stresses. By the continuous activity of osteoclasts and osteoblasts, the center of the callus is ultimately replaced by compact bone, creating a bone tissue similar to the original, unbroken bone. This remodeling can take several months, and the bone may remain uneven for years.

Although bone is a highly vascularized tissue and can repair itself, the ability of a bone to heal naturally diminishes when the defect exceeds a critical size—generally considered to be 2 to 2.5 times the diameter of the bone—which typically does not heal spontaneously [10]. This introduces complexities in treatment, necessitating more than just natural healing processes. Large bone defects and injuries caused by accidents or old age are serious problems in orthopedics. Clinical interventions are required to treat fractures that are not able to self-heal. Clinically, bone grafting is performed to repair the damaged bones and involves a surgical procedure that uses bone tissues of similar materials to rebuild the damaged bones. Autografts and allografts are the common treatment options, and the former is regarded as the ‘gold standard’ procedure to heal bone [3]. The advantages of autografts are that they are osteogenic, have histocompatibility, can provide structural support, and reduce the risk of disease transmission. In contrast, the drawbacks are their limited availability, blood loss, the requirement of anesthesia, donor site morbidity, infection, prolonged wound drainage, reoperation, and pain [16].

## 2. Criteria for the Selection of Scaffold Biomaterials

**Figure 4:**
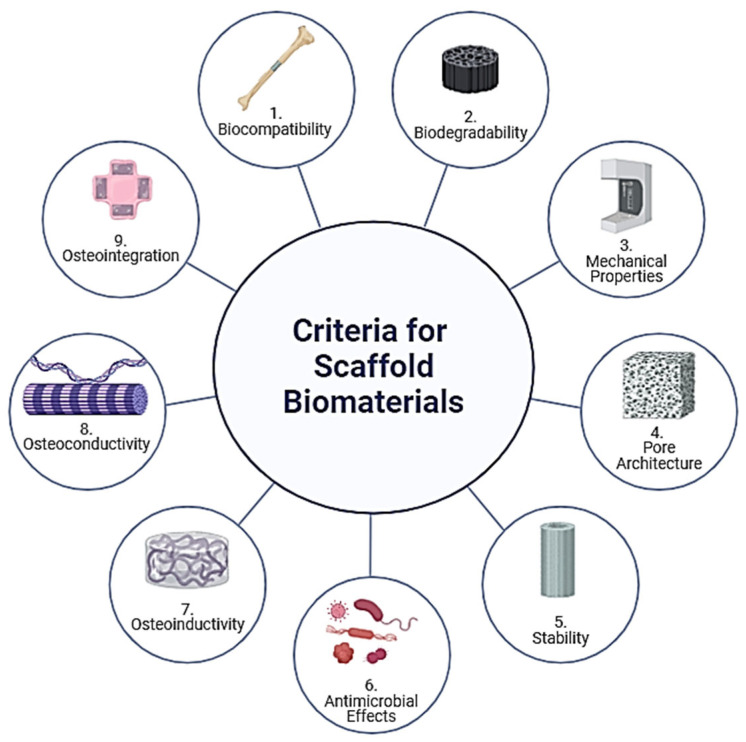
Desired properties in scaffold fabrication.

The selection of biomaterials used for the scaffold is of key importance. Figure 4 shows the desired properties of a scaffold: biocompatibility, biodegradability, mechanical properties, pore architecture, stability, antimicrobial effects, osteoinductivity, osteoconductivity, and osteointegration [17]. Research has focused on optimizing these scaffolds to support cell growth, differentiation, and eventual tissue integration.

1. Biocompatibility

Biocompatibility ensures that scaffolds do not elicit a harmful immune response upon implantation. Materials must interact favorably with the body’s biological environment, supporting cell attachment, proliferation, and differentiation without inducing cytotoxicity or inflammation. Recent studies also explore the scaffolds’ role in immunomodulation—designing materials that can positively interact with immune cells to enhance healing and integration [18]. Innovations include surface modifications and the integration of bioactive signals that can direct immune cell responses to favor regenerative processes.

In order to call a material biocompatible, it must facilitate the integration of new bone tissue with the existing bone (osteointegration) and support the formation of new bone tissue (osteogenesis). Materials like HAp and tricalcium phosphate are favored for their bioactive and osteoconductive properties, promoting bone cell attachment and growth [17]. Additionally, scaffolds can be functionalized with bone morphogenic proteins (BMPs) and other growth factors to enhance osteoinductivity—inducing stem cells to differentiate into osteoblasts, thus fostering bone regeneration [17,19].

2. Biodegradability

Biodegradability is essential for scaffolds as it allows them to gradually disintegrate, giving way to newly formed tissue. The rate of degradation should match the rate of tissue formation to ensure structural support during the healing process and to minimize chronic inflammatory responses caused by material remnants. Materials such as poly(lactic-co-glycolic acid) (PLGA) and polycaprolactone (PCL) are commonly used due to their known degradation rates and by-products that are safely absorbed or excreted by the body [20,21]. Control over the scaffold’s biodegradation can be achieved through the manipulation of polymer blends, molecular weights, and copolymer ratios, enabling customization for specific tissue targets [22].

3. Mechanical Properties

The mechanical properties of scaffolds, including stiffness, elasticity, and tensile strength, should mirror those of the target tissue to ensure functional integration and to support physiological loads. This is particularly important in tissues subjected to dynamic mechanical environments, like bone and cardiac tissue. It is preferable for scaffolding materials to have comparable values of bending strength and elastic modulus, which typically range from 100 to 150 MPa and 7 to 25 GPa for human cortical bone, respectively [23]. 

Scaffold mechanics can be tailored through material selection, structural design (e.g., fiber alignment, pore size), and fabrication techniques, such as 3D printing, which allows for precise control over the scaffold architecture. It has been documented that the mechanical properties of scaffolds are inversely proportional to their porosity, whereby a porosity of 200–350 µm is deemed appropriate for scaffolds used in the regeneration of human bone tissue [17,24,25].

4. Pore Architecture

Optimal pore size and porosity are critical for nutrient and oxygen diffusion, waste removal, and vascularization—all of which are vital for cell survival and proliferation within the scaffold. The interconnectivity of these pores also facilitates the integration of the scaffold with surrounding tissue by enabling cell migration and the formation of a new extracellular matrix. Techniques such as gas foaming, freeze drying, and particulate leaching are employed to create scaffolds with specific porosity and interconnectivity tailored to the needs of different tissues [17,22].

5. Stability

Chemical and physical stability in physiological conditions is crucial for maintaining the scaffold’s structural integrity until the tissue is fully regenerated. Stability is influenced by the chemical composition of the materials used and their resistance to biodegradation and environmental stressors, such as pH and enzymatic activities. Advanced scaffold designs incorporate crosslinking agents and composite materials to enhance stability without compromising biocompatibility or functionality [22].

6. Antimicrobial Effects

Scaffolds may be designed with antimicrobial properties to prevent infections, a common complication in implant surgeries. This can be achieved through the incorporation of antimicrobial agents, such as silver nanoparticles, or through surface modifications that resist bacterial adhesion and proliferation [26,27,28]. These properties are especially crucial in scaffolds used for skin, dental, and bone tissue engineering, where the risk of infection is significant.

### 2.1. Polymers

Scaffold biomaterials are the basic components of scaffolds that closely mimic the natural bone ECM and can be naturally derived or synthetic polymers. Polymers are long-chain organic materials joined together by covalent bonds. The most commonly used natural polymers for BTE include collagen, chitosan (CS), alginate, and silk; synthetic polymers include PCL, polylactide (PLA), poly(L-lactic acid) (PLLA), and poly(lactic-co-glycolic acid) PLGA [29,30,31,32,33,34,35,36]. For instance, collagen, the most abundant protein in bone, provides natural scaffolding properties that facilitate cell adhesion and migration, essential for tissue regeneration [37]. CS is recognized for its biocompatibility and antimicrobial properties, while fibrin, an integral component of the blood clotting cascade, serves as a natural scaffold, promoting cellular interactions and tissue development. Polymer-based scaffolds are highly porous, which enhances vascularization but reduces mechanical properties compared to bone [38,39].

Compared to natural polymers, synthetic polymers are more tunable in terms of modulus, strength, and biodegradation rate, and can be manipulated chemically to adapt the materials’ properties (such as degradation and structure) to the specific application requirements. PLA and PGA are favored for their mechanical robustness and moldability, which allow for the creation of scaffolds with precise architectures tailored to specific anatomical needs. These polymers are particularly advantageous in applications requiring gradual degradation and replacement by natural bone tissue. PLA provides a slower degradation rate, suitable for long-term support, whereas PGA offers a faster resorption rate, useful in scenarios where quicker scaffold replacement by natural tissue is desired [40,41]. However, the downside of synthetic polymers is that the degradation by-products can sometimes be toxic. 

Natural or synthetic polymers are required to crosslink to ensure mechanical stability under physiological conditions. The degree of crosslinking determines the mechanical strength and degradation rate of the polymer; the polymer initiator or the crosslinking agent added to crosslink the polymeric materials should not cause cytotoxicity to the living cells. Growth factor molecules and cells can also be loaded into the polymer for immediate commencement of bone healing [42,43]. 

### 2.2. Ceramics

Ceramics are inorganic materials used as bone tissue scaffolds. Earlier bioinert ceramic materials, such as alumina and zirconia, are used as implants [44,45]. However, these have been almost totally replaced by bioactive ceramics, such as HAp, calcium sulfate, tricalcium phosphate, and bioactive glasses. Calcium phosphate-based HAp is the main component of bone, and biomaterials based on this composition are the most common type of bioactive ceramics [46]. These materials exhibit high biocompatibility, promoting both osteoconduction and osteoinduction. Moreover, they possess suitable mechanical properties and can release calcium and phosphorous to support new tissue growth, although their degradation rates are not as controllable. However, a significant drawback of ceramic materials remains their brittleness.

### 2.3. Metals

Titanium and magnesium and their alloys are most commonly used in BTE, mainly for their high mechanical performance and durability. However, they are not bioactive materials and do not actively participate in bone healing. They also risk corrosion and fatigue over time. Titanium is valued for its exceptional strength, biocompatibility, and resistance to corrosion. It does not interact significantly with the biological environment, which minimizes the risk of rejection and allergic reactions. Titanium and its alloys, such as Ti-6Al-4V, are often used for permanent implants in load-bearing areas due to their high fatigue strength and excellent mechanical properties [47]. However, their inert nature means they do not degrade within the body, requiring surgical removal if not intended as a permanent solution. Advanced surface engineering techniques, such as the application of bioactive coatings and surface texturing, are being explored to enhance the osseointegration of titanium implants [47].

In contrast, magnesium is biodegradable and has mechanical properties closer to those of natural bone, which reduces the risk of stress shielding—a condition where implants can bear too much load, inhibiting natural bone growth [48]. Magnesium alloys are promising for temporary implants as they naturally degrade in the body’s environment. This degradation can stimulate new bone growth by releasing beneficial ions, such as magnesium, which may enhance bone formation. However, controlling the rate of degradation is critical to prevent rapid disintegration and loss of mechanical integrity before adequate bone healing. Current research focuses on alloying magnesium with elements like calcium or using coating technologies to control its corrosion rate, tailoring its properties for specific biomedical applications [48]. Particularly, the development of magnesium scaffolds through surface modification techniques, such as sol–gel and plasma spraying, has shown promising results in enhancing bioactivity and mechanical properties, critical for load-bearing applications. These scaffolds are designed to degrade at a controlled rate, matching the new tissue formation, thus eliminating the need for scaffold removal surgeries [49,50,51].

### 2.4. Composites and Hybrid Materials

The key points to consider while designing an ideal bone tissue-engineered scaffold are that the biomaterial chosen should be biocompatible and mimic the natural bone extracellular matrix. The material should be osteoinductive and recruit osteogenic cells that can differentiate into phenotypically desirable type cells based on the morphogenic signals. The scaffold should be sufficiently capable of vascularization to meet the growing need for nutrient supply as the bone cells develop. There is no such thing as a universally biocompatible material; the support material must be chemically designed to maximize its biocompatibility. 

One of several methods used to improve biocompatibility is to combine or blend different materials to form a composite material. The idea behind such composites is that by combining two or more constituents with different physical or chemical properties, the processability, mechanical properties, bioactivity, etc., of the final product will be enhanced compared to their individual constituents. A common type of composite used in BTE is the polymer matrix composite, which is a combination of biodegradable polymer and bioceramics particles. The bioceramic particles, usually in the form of nanoparticles, are dispersed into the polymer phase. Polymers have low mechanical properties compared to ceramics; but by adding ceramic particles into the polymer matrix, the synthesized material can have better mechanical and biological properties than if used individually. For example, Pádua et al. [52] synthesized a composite scaffold by combining CS polymer, mesoporous HAp, and bioactive glass ceramics. They compared the bioactivity and mechanical properties of the polymer, polymer–HAp composite, polymer–bioglass composite, and polymer/HAp/bioglass composite. Overall the composite CS/(75%) bioglass and CS/(50%) ceramics performed better than other combinations. Table 1 summarizes studies that use a combination of the CS polymer with other materials such as HAp, bioglass, and other additives to improve the mechanical properties and bioactivity of scaffolds.

Another article by Khan et al. [53] developed a bone tissue scaffold using a combination of arabinoxylan, B-glucan, nano-HAp, graphene oxide, and acrylic acid by free-radical polymerization, and the porous scaffold was then coated with CS to improve the biological activities. The addition of nano-HAp and graphene oxide improved the mechanical and biological properties of the composite. Polymers such as PLA are excellent biomaterials for BTE, but low osteoconductivity and acidic degradation limit its usage. The limiting factor of polymers has been shown to be improved by the addition of osteoconductive calcium carbonate and β-tricalcium phosphate [54]. The acidic degradation products were counteracted by the presence of calcium carbonate and maintained a neutral pH. The polymer–ceramic composite has a synergistic effect, low flexibility and plasticity for ceramics, and for polymers, it has low osteoconductivity and mineralization; when combined, these limits are reduced. Wu et al. fabricated composite scaffolds by reusing natural resources [55]. The composite was synthesized with HAp extracted from fish teeth, gelatin from fish scales, magnesium oxide, and polybutylene succinate. Magnesium oxide particles were encapsulated within gelatin in order to have a slow release of Mg^2+^ ions, and these ions improved the osteoblast cells’ proliferation. The composite also showed to have better tensile strength and anti-bacterial properties. 

While fabricating the polymer–ceramic composite, the ceramic particles should be well dispersed throughout the polymer but, often, the ceramic particles tend to migrate within the structure and form agglomerates. This can adversely affect the structural stability and biological properties of the composite material. Binding the bioactive ceramic particles to the polymers inhibits their movement. Łańcucka et al. modified the surface of silica particles with amino acids and covalently attached them to the collagen/CS/hyaluronic acid polymer phase via a genipin crosslinker, offering a homogeneous distribution of silica particles and avoiding phase separation [56].

**Table 1:** Summary of studies that combine CS with other additives to improve the mechanical properties and bioactivity of scaffolds.

Study Ref.	Material Composition	Method Used	Key Findings	Compressive Strength	Bioactivity
[57]	CS/nano-HAp composites (30/70 weight ratio)	Co-precipitation	High biodegradability and bioactivity; best ratio for mechanical strength and bioactivity	120 MPa	High in simulated body fluid (SBF) solution
[58]	CS hydrogel/HAp	Wet chemical synthesis	Enhanced biocompatibility with MG-63 osteosarcoma cells	-	-
[59]	CS/silica/HAp/Ca-GP	Sol–gel	High cell proliferation and growth; promising composite for filling small bone defects	0.3 to 10 MPa	-
[60]	CS/nano-HAp	In situ combination	Improved bioactivity with pre-osteoblasts; nano-HA content positively affects bioactivity	-	Enhanced mineralization
[61]	CS/nano-HAp	In situ hybridization	Enhanced mechanical properties; suitable for scaffold applications with homogenous nanoparticle distribution	Acceptable for tissue substitution	Homogeneous integration
[23]	CS/HAp/magnetite nanocomposites	Mixed composites	Superior mechanical properties due to magnetite addition	-	In vitro biocompatibility
[62]	Hydroxypropyl chitosan (HPCS)/nano-HAp	Genipin crosslinking	Improved mechanical properties and cell mineralization; effective for osteogenic potential and scaffold stability	-	Enhanced alkaline phosphatase (ALP) activity
[63]	CS–HAp/PMMA	Freeze drying and radical polymerization	Stable thermal properties; favorable outcomes for cell population and spreading in vitro; addition of PMMA significantly improved mechanical strength	-	Non-toxic to cells
[64]	CS/HAp/β-TCP composites	Crosslinking with TPP	Improved mechanical properties and lower biodegradation; ideal for high-load-bearing bone applications	4 kPa to 17 kPa	2% lower biodegradation with a higher HAp/β-TCP ratio

## 3. Scaffold Fabrication Techniques

Tissue-engineered synthetic bone substitutes were developed to overcome the limitations of autografts and allografts. The bone substitute, usually in the form of scaffolds produced in a lab, can be introduced to the bone defect site, which aids in bone remodeling. These artificial scaffolds act as a three-dimensional (3D) bone ECM and support the attachment of cells, promote the deposition of minerals, and provide mechanical support. An ideal scaffold should be biocompatible and biodegradable and should support vascularization. The three main elements of BTE are appropriate scaffold biomaterials, stem and precursor cells, and growth factors. BTE most often incorporates porous 3D scaffolds along with cells and bioactive growth factors, providing structural support for cells to spread, migrate, and differentiate—requirements to promote new tissue formation. 

Stem cells, such as MSCs, induced pluripotent stem cells (iSPCs), and embryonic stem cells (ESCs), are foundational to BTE due to their self-renewal capabilities and potential to differentiate into various tissue types. MSCs are multipotent stromal cells that can differentiate into osteoblasts, chondrocytes (cartilage cells), and adipocytes (fat cells). They are commonly harvested from bone marrow, adipose tissue, or umbilical cord blood. MSCs are favored for their osteogenic potential for bone formation. They are often seeded onto bone scaffolds where they can differentiate into bone cells and aid in the deposition of new bone tissue. Additionally, MSCs have immunomodulatory properties that are beneficial for reducing inflammation at the site of implantation [65]. iSPCs are derived by reprogramming somatic cells to an embryonic-like pluripotent state, allowing them to differentiate into almost any cell type. In BTE, iPSCs are particularly valuable because they can be sourced from the patient, reducing the risk of immune rejection [66]. They can differentiate into osteoblasts, chondrocytes, or even endothelial cells, depending on the scaffold’s requirements. When incorporated into scaffolds, iPSCs can contribute to the formation of a new bone matrix, vascular structures within the scaffold, or both. Thus, iPSCs are considered a powerful tool for creating complex, multi-tissue constructs that mimic the natural bone environment. ESCs are derived from early-stage embryos and are pluripotent in nature, allowing them to differentiate into all three germ layers and offering great potential for generating any cell type required for tissue generation [67]. When embedded within scaffolds, ESCs can be directed to form not only bone but also supportive vascular and connective tissues in the development of complex, integrated tissue structures. Their pluripotency, combined with their high proliferative ability, makes ESCs a powerful but ethically sensitive option in scaffold fabrication due to their acquisition from embryos [68]. 

Growth factors include VEGF, bone morphogenetic proteins (BMPs), insulin-like growth factors (IGFs), transforming growth factor-beta (TGF-β), and fibroblast growth factors (FGFs). These aid in directing cells to their phenotypically desirable type and enhancing their growth. BMPs are a group of growth factors also known as cytokines and are a part of the TGF-β superfamily. They are particularly significant in BTE because they have been shown to induce the formation of bone and cartilage. VEGF is paramount for angiogenesis. In scaffold fabrication, promoting angiogenesis is crucial for the survival of newly formed tissues, particularly in avascular regions, such as certain bone segments. BMPs are often incorporated for their role as signaling molecules to enhance osteoinductivity—encouraging MSCs to differentiate into osteoblasts [69]. IGFs, mainly IGF-1 and IGF-2, are involved in anabolic processes and are crucial for bone growth and development. IGFs can promote the proliferation and differentiation of osteoblasts and are also important for angiogenesis, which is critical for scaffold integration and survival in vivo [70,71]. TGF-β is involved in cell growth, differentiation, and healing. TGF-β can help modulate the immune response to scaffolds and is also involved in the chemotaxis and differentiation of cells necessary for tissue regeneration [71]. It plays a role in both the formation of new bone and the repair of damaged bone tissue. FGFs are involved in angiogenesis, wound healing, and embryonic development [72]. FGFs can stimulate the proliferation of endothelial cells and fibroblasts, which are essential for creating a supportive matrix in BTE. In scaffolds, FGFs can be used to stimulate vascularization, ensuring that engineered tissues receive sufficient nutrients and oxygen.

The design and development of scaffolds aim to replicate the complex architecture and multifunctional nature of the bone ECM. This involves supporting mechanical loads as well as facilitating cellular activities essential for tissue regeneration and integration. The sophistication of scaffold design has significantly progressed with the advent of advanced fabrication techniques that allow for precise control over scaffold properties at the micro- and nanoscales.

### 3.1. Additive Manufacturing (3D Printing)

The preparation of the scaffold requires precise control of the scaffold material. Its structure should be porous, with interconnected pores, and its size should be appropriate for efficient mass transport. The fabricated structure should fit perfectly, completely matching the anatomical structure of the bone defect, and should also mimic the mechanical and biological properties of native bone. Traditional methods such as solution casting, electrospinning, and lyophilization are not able to precisely control the fabrication process and are unable to completely achieve the aforementioned criteria of bone scaffolds. To overcome these drawbacks, 3D printing methods were developed for BTE, shown particularly effective in the treatment of complex bone defects seen in craniomaxillofacial operations and other specialized bone defects [3]. Three-dimensional printing, or additive manufacturing, is a rapid prototyping technology capable of customizing and fabricating scaffold components by depositing the materials layer by layer (LbL) using computer-aided design (CAD) and computer-aided manufacturing (CAM) systems. For instance, recent developments in 3D bioprinting have utilized various biomaterials to create scaffolds that support vascularization and robust cell growth [73,74,75]. The incorporation of bioactive ceramics and advanced bioprinting technologies, such as core/shell bioprinting, has led to the fabrication of hybrid scaffolds that offer enhanced mechanical properties and biological functionality [76]. Table 2 summarizes recent studies that demonstrate the capabilities and advancements of 3D printing techniques in scaffold fabrication.

Incorporating electrospinning, molecular self-assembly, and phase separation into additive manufacturing is one other technique that has demonstrated success in achieving highly porous structures with interconnected pores for efficient mass transport and cell proliferation [3]. This method has been shown to enable the production of scaffolds with drastically enhanced surface area, surface roughness, and surface-area-to-volume ratios by shrinking the material size to the nanoscale. These modifications result in superior physicochemical characteristics that significantly influence the scaffold’s osteoinductivity and osteointegration through enhanced nanotopography. 

For example, combining 3D printing with electrospinning has enabled the creation of composite scaffolds that not only meet the mechanical and structural requirements of the native bone but also support enhanced vascularization, essential for the healing of complex bone defects [76]. Electrospinning has also been utilized in conjunction with 3D printing to produce fibrous scaffolds, such as those made from silica, using materials such as tetraethyl orthosilicate (TEOS) and polyvinyl alcohol (PVA). These scaffolds exhibit superior physicochemical characteristics, promoting bioactivity and osteointegration [83]. The fibrous structure mimics the ECM’s fibrous component, providing an ideal environment for cell attachment and differentiation.

The LbL technique, a form of molecular self-assembly, has been leveraged to create nanolayer films that provide controlled bioactivity and mechanical stability, tailored for osseous defect therapies [84]. This method allows for precise surface and bulk modifications of scaffolds, enhancing their integration with native bone tissue. Furthermore, nano-featured scaffolds, which act as temporary and synthetic ECM replicas, support cell attachment and guide three-dimensional bone tissue formations [3,85]. The main constituents of bone ECM are in the nanoparticle range, and it has been well-established that native bone cells interact well with nano-sized proteins and minerals.

Phase separation, particularly thermally induced phase separation (TIPS), has emerged as a sophisticated method for creating 3D-printed scaffolds with precise control over porosity and microarchitecture. This technique enables the fabrication of scaffolds with highly interconnected porous networks, ideal for promoting efficient mass transport, nutrient diffusion, and enhanced cellular infiltration. A study by Sultan et al. demonstrated that using TIPS to incorporate bioactive glass particles into polylactic acid (PLA) matrices significantly enhances the mechanical properties of the scaffolds while maintaining excellent biocompatibility and promoting osteointegration [86]. 

The adaptability of the nonsolvent-induced phase separation (NIPS) process to incorporate various biomaterials has been showcased in another study by Aydin et al., where a composite of PCL and nano-HAp was developed [87]. The study reported that scaffolds fabricated via NIPS exhibited superior pore interconnectivity and uniformity, which are critical for facilitating cell growth and the formation of new bone tissue. These scaffolds showed enhanced mechanical strength and bioactivity, leading to improved osteogenic differentiation and mineral deposition. Notably, by combining phase separation with other nanostructuring techniques, scaffolds can be tailored to exhibit dynamic responses to physiological stimuli, enhancing their functionality in regenerative medicine applications.

### 3.2. Functionalization of Scaffolds

#### 3.2.1. Cell and Bioactive Molecule-Laden Scaffolds

Another strategy used to improve the design of the scaffold is to incorporate stem cells into its structure. Different types of cells are involved in bone formation and remodeling processes, such as osteocytes, osteoblasts, and osteoclasts. The most commonly used stem cells are adult MSCs, iPSCs, and ESCs, all of which can differentiate into bone-forming cells. Since cells are encapsulated, the scaffold fabrication must be performed carefully, and a proper supply of nutrients for the cells is required. Typically, the inclusion of cells is often facilitated by materials with high water content, such as hydrogels, which can envelop cells and provide a suitable environment for cell viability and differentiation. The scaffold can be seeded with coated cells a short time before the implantation, or the cells can be injected into the scaffold after the implantation. For example, an article by Heo et al. demonstrated the effective encapsulation of MSCs and human umbilical vein endothelial cells (HUVECs) in a collagen/fibrin hydrogel, leading to the differentiation of MSCs into osteogenic cells and the formation of HUVECs into pre-vascular networks [88]. 

Apart from stem cells, biologically active molecules such as growth factors, peptides, and pharmaceuticals can be entrapped or encapsulated into the scaffold materials to improve bone regeneration [89]. For instance, the concept of scaffold-induced cell homing enhances the previous approach by incorporating chemokines and other bioactive molecules that attract stem cells to the injury site. This process relies on biodegradable scaffolds strategically placed in the defect area to release these chemotactic agents, facilitating the mobilization and homing of MSCs. Critical molecules in this process include various mimetic peptide sequences like RGD (Arg-Gly-Asp), GFOGER (Gly-Phe-Hyp-Gly-Glu-Arg), YIGSR (Tyr-Ile-Gly-Ser-Arg), IKVAV (Ile-Lys-Val-Ala-Val), and REDV (Arg-Glu-Asp-Val). These peptides mimic natural ECM components, providing essential biochemical cues that promote cell attachment as well as improve osteoblast functionality and overall osteointegration within the host tissue [3]. Natural ECM proteins, such as collagens and fibronectin, have also been proposed for integration within scaffolds [90]. However, only a few short peptide sequences within these large macromolecules serve as integrin recognition and binding sequences that trigger subsequent processes like cell adhesion, signaling, and proliferation. The aforementioned oligopeptides are thus sufficient enough to convey bioactivity to implant materials. 

The RGD sequence promotes the attachment of various cell types by interacting with specific receptors on the surface of integrin [91,92,93,94,95]. It is immobilized on the surface of the scaffold to activate cell proliferation and regulate cell metabolism and ECM synthesis [96]. GFOGER, YIGSR, and IKVAV have been particularly identified from native bone ECM components and were reported to bind specific receptors on the integrins and activate intracellular signaling pathways [92,97,98,99,100]. GFOFER, a collagen derivative, has been utilized in several studies as a cell adhesion peptide [101,102]. Notably, GFOFER has demonstrated its capacity to support MSC differentiation into chondrogenic cells with increased expression and deposition of type II collagen and glycosaminoglycans, thereby displaying its potential as a mimetic ligand for osteogenic differentiation. Lastly, REDV specifically targets endothelial cells, enhancing their proliferation, and thus angiogenesis [37,103,104,105,106,107].

**Figure 5:**
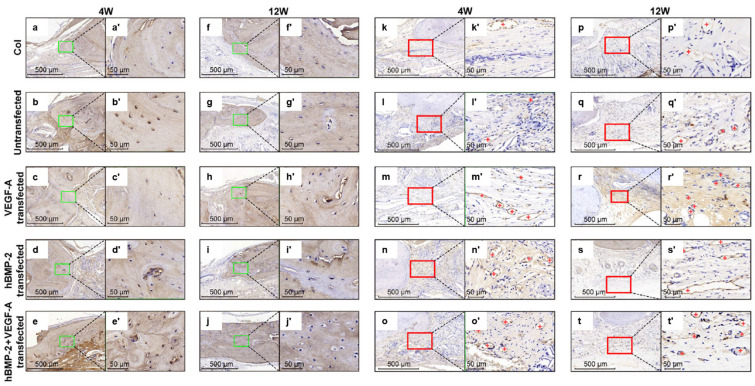
Photomicrographs indicating that BMP-2 and VEGF incorporation within scaffolds stimulates neobone formation for (**a**–**j**) OCN-stained bone tissue; (**a**–**e**) defect areas at 4 weeks and (**f**–**j**) at 12 weeks post-treatment; (**k**–**t**) CD31-stained blood vessels denoted by a red ‘+’; (**k**–**o**) defect areas at 4 weeks and (**p**–**t**) at 12 weeks post-treatment. Rectangular areas in (**a’**–**t’**) correspond to magnified areas in images (**a**–**t**). Adapted from [125], with modifications.

Moreover, growth factors are commonly incorporated into scaffolds to improve osteoinductive and osteoconductive properties. Research has shown that the incorporation of BMP, TGF-β, VEGF, and FGF into scaffolds significantly enhances the osteogenic differentiation of seeded cells and promotes the healing of bone defects [108,109,110,111,112,113,114,115,116,117]. These growth factors are embedded within the scaffold matrix and are released in a controlled manner, providing sustained stimulation to the surrounding cells. The controlled release is particularly critical as it mimics the natural healing process where growth factors are gradually made available at the site of injury or regeneration. In addition, VEGF has been extensively studied for its role in promoting vascularization within scaffolds, which is essential for supplying nutrients and removing wastes, thus supporting the survival and proliferation of newly formed tissue. The integration of VEGF into scaffolds has been shown to improve the formation of blood vessels, enhancing the overall regeneration of bone tissue [66,118]. Particularly, BMP and VEGF have exhibited a regulatory coupling effect between osteogenesis and angiogenesis in which the rapid early release of VEGF and the sustained slow release of BMP-2 are identified as optimal [119,120,121,122,123]. The effective incorporation and immobilization of BMP-2 and VEGF within multilayered polydopamine (PDA) coatings was achieved in a study conducted by Godoy-Gallardo et al. [124]. Specifically, BMP-2 and VEGF were bound to the inner and outer PDA layers, respectively, which resulted in their sequential adsorption as well as osteogenic and angiogenic synergy. Overall, advanced techniques, like LbL assembly, may be employed to allow for precise control over the spatial distribution of the growth factors within the scaffold. This method involves the sequential adsorption of polyelectrolytes and growth factors, forming multilayered coatings that can release bioactive molecules in a controlled manner. Figure 5 illustrates the results from a study conducted by Geng et al., which shows how the synergy between BMP-2 and VEGF can stimulate neobone formation [125]. It can be observed that the BMP-2 + VEGF treatment group yields a higher density of osteocalcin (OCN)-positive cells and blood vessels in the regenerating area—Figure 5j’,t’, respectively [125].

Several other advanced embedding techniques have been developed. Among these, encapsulation is one common technique, where growth factors are entrapped within biodegradable microspheres or nanoparticles dispersed throughout the scaffold matrix, allowing for a sustained release. Recent in vitro studies by Zhao et al. demonstrated that BMP-2 encapsulated in PLGA microspheres when integrated into a 3D printed PLGA/CaSO_4_ scaffold, significantly promoted osteogenic differentiation over an extended period [126]. 

**Figure 6:**
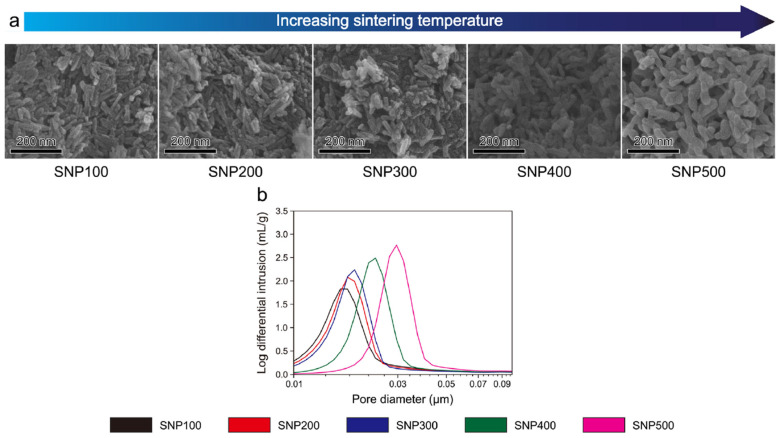
(**a**) SEM images showing increasing pore size with sintering temperature (100–500 °C); (**b**) internal pore size distributions. Adapted from [127], with modifications.

The microstructural characteristics of scaffolds, such as porosity, pore size, and interconnectivity, play important roles in the effective delivery of growth factors. On the one hand, the macro-porous and micro-porous structures of natural bones support osteogenic differentiation by aiding the spreading and elongation of stem cells [127,128,129]. Nano-porous structures, however, provide a large surface area for the adsorption of proteins, including growth factors [127,130,131,132]. These nanopores can also alter macrophage morphology by creating different immune environments and can induce the recruitment and differentiation of osteoblasts during the early stages of bone formation [127,133]. High porosity and optimal pore size are vital for cell migration and vascularization, while interconnected pores ensure the uniform distribution and gradual release of growth factors. Figure 6, observed through scanning electron microscopy (SEM), shows that increasing the sintering temperature affects the nanopore size in HAp scaffolds containing nanopores (SNPs) [127]. Kim et al. found that scaffolds sintered at the highest temperature of 500 °C, which produced the largest nanopores, significantly enhanced cell proliferation and differentiation rates [127]. Additionally, these larger nanopores (SNP500) facilitated more rapid liquid flow and greater protein adsorption. While the total surface area does indeed influence protein adsorption, pore size and liquid flow are the primary criteria for selecting porous scaffold structures [127].

#### 3.2.2. Surface Modification

Surface modification techniques in the development of orthopedic scaffolds represent another advancement in improving the integration and functionality of implants within diverse tissue environments. In the particular context of orthopedic tissue interfaces, tri-phasic layered scaffolds have been designed to address the diverse and complex structure of hard tissue–soft tissue interfaces, such as those between bone, ligament, and cartilage [3,134,135,136,137]. Surface modifications in these scaffolds are specifically engineered to optimize cell adhesion and integration at each tissue interface, effectively supporting the formation of integrated multi-tissue systems. This is achieved by adjusting the surface properties, such as hydrophilicity, roughness, and functional group presentation, which are crucial for enhancing cellular interactions and osteointegration.

## 4. Bio-Responsive Scaffolds

### 4.1. Physical Stimuli

Bio-responsive scaffolds are engineered to be sensitive to a spectrum of physical stimuli such as magnetism, temperature, ultrasonic waves, and magnetic fields. This responsiveness is not merely passive but enables a reconfiguration of the scaffold’s structure in situ, which is crucial for targeted therapeutic interventions. Particularly noteworthy are temperature-responsive scaffolds, which maintain structural integrity and functionality under physiological conditions yet are designed to undergo selective degradation when exposed to pathological environments typical of diseased tissues. Such capabilities suggest the potential of physical-responsive scaffolds to improve drug delivery systems, offering controlled release mechanisms that are finely attuned to the body’s varying biophysical cues.

#### 4.1.1. Temperature Response

Temperature-responsive scaffolds utilize materials, such as hydrogels and polymers, which respond to local temperature changes, making them highly effective for site-specific drug delivery and structural adaptation during the various stages of bone healing. Research by Durairaj et al. has demonstrated that thermosensitive hydrogels, which exhibit temperature-dependent gelation properties, transition from liquid to gel at body temperatures, effectively conforming to bone defect sites before solidifying [138]. This ability offers structural support and facilitates localized drug delivery. The study shows the potential of one such hydrogel, a methylcellulose (MC)-based hydrogel known for its osteoinductive properties, in delivering bioactive compounds, like veratric acid (VA), directly to inflamed or healing bone tissues. ALP, an enzyme commonly used as a marker of osteogenic differentiation, can indicate the maturation of cells into osteoblasts. The reported significant increase in ALP activity and enhanced osteogenic differentiation were primarily attributed to the thermal responsiveness of the hydrogel itself, as well as the presence of VA encapsulated within the CS/MC hydrogel matrix. While the improved cellular response was not directly induced by thermal stimuli, it was indeed facilitated by the scaffold material’s ability to function effectively at body temperature, ensuring that bioactive components, like VA, were maintained in an active state conducive to interacting with the cellular components. This interaction is critical for promoting osteogenic differentiation in MSCs, verified by increased calcium phosphate deposition, evident from von Kossa staining [138].

Further developments have been made by Woodbury et al. through the fabrication of scaffolds from materials like poly(ε-caprolactone-co-lactide) (PCL-PLLA) [110]. The nanofibrous scaffold demonstrates a critical partial-melting temperature at 52 °C, allowing for deformation at this elevated temperature and retention of structural properties upon cooling to 37 °C [139]. To improve its utility in BTE, incorporating bioactive molecules, such as BMPs, into the scaffold could support natural bone healing by releasing these growth factors in response to temperature-induced phase changes. Additionally, research into biocompatible phase change materials that operate at temperatures closer to body temperature could reduce thermal stress on surrounding tissues, increasing the scaffold’s safety and feasibility. Research by Vejjasilpa et al. explores a stimuli-responsive scaffold fabricated through continuous digital light processing (cDLP), which employs a resin formulation adjusting the phase transition temperature [140]. Notably, the scaffold’s phase transition temperature is adjustable based on the resin composition, enabling thermally induced mechano-stimulation of cells. This temperature is critical for applications requiring temperature responsiveness directly at physiological conditions. Table 3 summarizes the recent studies on temperature-responsive scaffolds.

Despite these advancements, achieving precise thermal responsiveness in scaffolds remains a considerable challenge due to the variability of pathological conditions within living tissues. Current research is thus directed towards developing materials capable of responding effectively at lower temperature thresholds to enhance their stability and safety in normal tissues.

#### 4.1.2. Mechanical and Ultrasound Response

Mechanical stimuli, such as pressure, stress, and strain, are ubiquitous in the physiological environment of bone tissues, which are naturally subjected to various mechanical loads. The use of piezoelectric materials in BTE has garnered interest due to their ability to convert mechanical stress into electrical signals, thereby promoting osteogenesis through electrical stimulation of bone cells [141,142,143,144]. Piezoelectric materials, such as polyvinylidene fluoride (PVDF) and its copolymers, when fabricated into fibrous scaffolds, demonstrate significant piezoelectric properties [6,142]. Under mechanical stress, these scaffolds generate electrical signals that mimic the natural bioelectrical signals in bone, enhancing the migration and differentiation of osteoprogenitor cells. Zheng et al. report the ability of piezoelectric materials to modulate cellular behavior by generating surface charges in response to deformation, offering new avenues for biomechanical simulation, bone regeneration, and bone defect repair [145]. The potential of such materials in BTE to enhance osteogenic differentiation through mechanical–electrical transduction pathways has also been highlighted [146]. 

A study by Miszuk et al. demonstrated that electrospun nanofibrous scaffolds made from PCL-HAp can effectively mimic the bone ECM and improve the scaffold’s mechanical strength, porosity, and elasticity, as well as maintain these properties after being evenly coated with minerals and pressed into varying defect shapes [147]. Moreover, the study explores the scaffold’s capability for localized, sustained drug release. They incorporated phenamil, a BMP-2 signaling agonist, using a bio-mimetic mineral deposition technique that allowed for the simultaneous encapsulation of various drugs under physiologically mild conditions. Compared to traditional scaffolds with surface-adsorbed phenamil, these composite scaffolds showed a reduced initial burst release and extended the duration of sustained drug release, enhancing the osteogenic differentiation of cells in vitro.

In addition to mechanical stimuli, ultrasound waves, particularly low-intensity pulsed ultrasound (LIPUS), can induce micro-vibrations within scaffold materials, which, in turn, promote essential cellular activities for bone healing. Research has shown that LIPUS can increase the callus’ mechanical strength, reduce time to bone union, promote osteocalcin mRNA expression in human osteoblasts, induce osteoblasts to release cytokines, regulate TGF-β, and influence all major cell types involved in bone healing [148,149,150,151]. He et al. have shown that sinusoidal continuous wave ultrasound can cause the responsive scaffold to resonate and produce centripetal acoustic radiation force, which can promote the adhesion and growth of bone marrow stem cells (BMSCs) on the surface of the scaffold and osteogenic differentiation [152]. The ultrasound treatment group exhibited better bone defect repair effects. Furthermore, Ambattu et al. experimentally demonstrated that short-duration, high-frequency acoustic vibration can induce the directional differentiation of BMSCs into osteoblasts [153]. These studies highlight the potential of using various forms of ultrasound to enhance the physical properties of scaffolds, i.e., mechanical strength and permeability for enhanced drug delivery of nutrients and therapeutic agents, and to directly stimulate cellular activities and differentiation [154]. Table 4 summarizes the recent studies on mechanical- and ultrasound-responsive scaffolds.

#### 4.1.3. Electrical Response

Electroactive materials, such as conductive polymers and piezoelectric components, are increasingly integrated into scaffolds to exploit their electrical properties for stimulating bone growth. Capacitive biomaterials capable of storing electrical charge on their surfaces, as well as conductive polymers like polypyrrole, polyaniline, and polythiophene, have shown promising results. Electrical stimuli also prompt the migration, proliferation, and differentiation of bone cells at specific sites in vitro, as well as boost healing via the interactions between bioelectrics and charged biomolecules [156,157,158,159,160,161]. Piezoelectric ceramics, such as barium titanate, BaTiO_3_ (BT), have exhibited high mechanical performance and modulus of elasticity close to that of native bone tissue [162,163]. However, their high brittleness and low damage tolerance restrict their processing flexibility and application in BTE to a certain degree [163]. A study by Jiao et al. shows that the integration of BT/HAp composite scaffolds provides an active response to mechanical deformation by generating electrical charges. The piezoelectric coefficient (d33 value) of the bulk ceramic composites and dry bone were measured at 0.61 pC/N and 0.7 pC/N, respectively [164]. This property is critical as it suggests that the material can effectively mimic the natural electrical behavior and piezoelectric response of bone. This modality of treatment is being further integrated with electric-responsive stents and is considered a compelling approach in clinical practice to expedite the bone healing process [6,165,166].

The development of electroactive scaffolds also involves innovative engineering approaches, such as the use of microfabrication techniques, to create patterns and structures that optimize the electrical properties of the scaffold. These techniques allow for precise control over the scaffold’s architecture, ensuring that the electrical signals are delivered in a manner that closely replicates the natural bioelectrical environment of bone tissues. This level of control is important for achieving targeted stimulation and enhancing the efficacy of the scaffold in supporting bone regeneration. Electrical stimulation is particularly effective in treating non-healing bone defects or where biological cues alone are insufficient to induce adequate healing. By applying controlled electrical signals, these scaffolds can activate various biological pathways that are vital for bone growth and repair. The ability to modulate these signals based on the changing requirements of the healing bone further highlights the adaptability and potential of electrically responsive scaffolds in clinical settings. Table 5 presents a summary of recent studies on electrical-responsive scaffolds.

#### 4.1.4. Magnetic Response

The development of magnetic biomimetic scaffolds involves integrating magnetic nanoparticles or coatings into scaffold materials, which can be manipulated externally by magnetic fields, including alternating magnetic fields (AMFs), which periodically change direction, as well as constant magnetic fields, which remain in one direction (CMFs) [6,167]. Fernandes et al. explored the integration of CoFe_2_O_4_ (CFO) magnetostrictive particles into a PVDF matrix, which mainly crystallizes in the electroactive β-phase of PVDF under the influence of magnetic stimuli, promoting the proliferation of pre-osteoblasts [168]. This transformation can be linked to the induction of electro-transduction processes through magnetoelectric responses of the scaffolds. The findings revealed that the application of a controlled magnetic field induced a significant transformation in the scaffold’s microstructure and led to an enhanced β-phase crystallization of PVDF [168]. This phase is known for its superior piezoelectric properties, which are crucial for stimulating osteogenic cell types. The study further demonstrated a marked increase in the proliferation and maturation of osteoprogenitor cells, indicating the scaffold’s potential to support rapid bone regeneration [168].

Paltanea et al. report on the use of biodegradable magnetic scaffolds composed of CS and PCL infused with magnetic nanoparticles (MNPs) (typically Fe_3_O_4_) [17]. One such study by Zhang et al. developed 3D-printed magnetic mesoporous bioactive glass (MBG)/PCL/Fe_3_O_4_ composite scaffolds that exhibit improved proliferation, alkaline phosphatase (ALP) activity, and upregulation of osteogenesis-related gene expressions (RUNX2, OCN, BSP, BMP-2, and Col-1) in human BMSCs [169]. Additionally, the scaffolds facilitated a sustained release of the anticancer drug doxorubicin (DOX), showcasing their potential for localized drug delivery even in oncological applications [169]. 

In a separate study, Lanier et al. developed a unique magnetic-responsive scaffold comprising PCL microparticles that encapsulate MNPs and placental proteins. The design leverages the magnetocaloric effect of the MNPs to induce localized heating. When exposed to an alternating magnetic field (AMF) ranging in strength from -1 to 1 Tesla, the induced heating causes the PCL to melt, facilitating the controlled release of embedded proteins [6,170]. This process not only supports bone formation but also allows for the scaffold to solidify again once the magnetic field is deactivated. Such a feature provides the intriguing potential for the cyclic, repeated administration of therapeutic agents, offering a sophisticated approach to localized, controlled drug delivery. This capability to precisely control the release profile enhances the scaffold’s application in promoting bone regeneration, illustrating the synergistic potential of combining magnetic functionalities with biodegradable polymers for advanced therapeutic strategies in bone healing and tissue engineering [170].

Despite these advancements, challenges persist in the practical application of magnetic scaffolds in clinical settings. The primary concern is based on the long-term biocompatibility and safety of MNPs, especially given their potential to diffuse from the scaffold and impact surrounding tissues. Some studies have indicated potential oxidative stress and inflammatory responses triggered by iron oxide nanoparticles, necessitating rigorous in vivo evaluations to establish safe usage parameters [171,172,173,174]. Table 6 summarizes recent studies on magnetic-responsive scaffolds.

### 4.2. Chemical Stimuli

The goal of chemical stimuli-responsive scaffolds in BTE is based on their ability to adapt their behavior in response to specific chemical signals. For instance, pH-responsive scaffolds are engineered to change their physical properties or release therapeutic agents when exposed to the acidic environments typically found in inflamed tissues. This ability allows for a more controlled and localized treatment approach, aligning the scaffold’s function with the physiological needs of the tissue. Similarly, scaffolds responsive to oxidative stress markers, like ROS, are designed to respond to the elevated ROS levels that are indicative of oxidative cellular environments in damaged bone tissues. These scaffolds can either release growth factors or degrade in a controlled manner, thus aiding the regeneration process by matching the release of therapeutic agents with the intensity of oxidative stress.

#### 4.2.1. pH Response

The design of pH-responsive scaffolds targets the natural variability in pH levels found in pathological states, particularly in areas with inflammation where pH can notably decrease. These scaffolds are engineered to respond to such changes, enhancing drug delivery and tissue regeneration by adapting to the biochemical shifts within the injury site.

Recent studies have explored various materials capable of responding to pH shifts, facilitating targeted therapeutic interventions. For instance, Zeolitic imidazolate framework-8 (ZIF-8), a class of metal–organic frameworks (MOFs), has been highlighted for its pH-sensitive properties, which make it an excellent candidate for bone substitution and as a drug delivery carrier [175,176,177]. ZIF-8 can effectively release Zn^2+^ ions in acidic environments, which are beneficial for bone regeneration due to their osteogenic impact [178]. This capability was demonstrated in a study where electrospun PCL/collagen membranes modified with ZIF-8 released a significant amount of Zn^2+^ ions under acidic conditions (pH 5.5), enhancing vascularized bone regeneration in a rat model with calvarial defects [179]. Comparing the use of ZIF-8 in different scaffold matrices provides insight into how scaffold composition can influence drug release kinetics and biological outcomes. For example, ZIF-8 nanocrystals have also been used as carriers for vancomycin (Van), demonstrating a controlled release of CS fiber scaffolds that not only delivered the antibiotic effectively but also promoted osteoblast differentiation by maintaining an acidic pH conducive to bone healing [6,180,181]. This illustrates the dual function of pH-responsive scaffolds in providing both antimicrobial protection and supporting bone regeneration. Table 7 summarizes recent studies on pH-responsive scaffolds.

#### 4.2.2. Redox Response and Reactive Oxygen Species (ROS)

Redox-responsive materials are specifically engineered to respond to the reactive oxygen species (ROS) prevalent in inflammatory and regenerative environments. These ROS include the superoxide anion (O_2_^−^), hydroxyl radicals (·OH), hypochlorite ion (ClO^−^), and hydrogen peroxide (H_2_O_2_), which play pivotal roles as signaling molecules in the progression of inflammatory disorders and are critically involved in bone growth and remodeling [183]. Redox-responsive scaffolds rely on the dynamic redox environment of healing bone tissues, where oxidative stress is naturally elevated, to modulate scaffold behavior.

In inflamed tissues, ROS levels can be significantly higher—up to 100 times—than in healthy tissues [184]. This marked increase makes ROS ideal targets for responsive scaffold systems designed to enhance bone healing processes. A notable study by Martin et al. explored the use of thioketal-based polymers within scaffolds that degrade upon encountering elevated ROS levels, thereby facilitating the localized release of bone morphogenetic protein-2 (BMP-2) [185]. This responsive degradation ensures that therapeutic agents are released in a controlled manner precisely where needed, significantly promoting bone regeneration in areas with critical-sized bone defects.

A study by Lee et al. utilized PLGA-based nanoparticles, demonstrating their potential in managing ischemia/reperfusion (I/R) injury, known for inducing ROS, like H_2_O_2_, which contributes to vascular thrombosis and tissue damage [186]. Heparin and glutathione were both encapsulated for their anticoagulant and antioxidant properties. This design facilitated controlled drug release, with a notably low percentage of heparin (10.3%) released over an extended period of 96 h, which is crucial for sustained therapeutic effects in vascular therapy. Furthermore, an H_2_O_2_-responsive platform was introduced by combining silk fibroin with horse peroxidase, enabling the detection of H_2_O_2_ and thus tailoring the therapy to oxidative stress levels observed in I/R injuries [186]. The nanoparticles were surface modified with hyaluronic acid to specifically target human BMSCs. The targeted delivery to hBMSCs enables these cells to uptake the therapeutic nanoparticles efficiently, within 2 h, with exocytosis observed 6 h post-uptake. This precision in delivery is particularly advantageous in BTE, where the modulation of ROS levels can significantly influence bone healing processes. The scaffolds’ ability to respond dynamically to the oxidative environment can both mitigate inflammation and improve the osteogenic capacity of the scaffold by supporting the survival and proliferation of bone-forming cells under stress conditions. The study thus presents a significant advancement in the development of ROS/redox-responsive scaffolds, offering a multifunctional platform that could be effectively utilized to treat vascular and bone-related diseases through controlled delivery and smart response mechanisms. 

The broader implications of ROS-responsive scaffolds in effectively managing drug release across various pathological tissues are profound. Variations in ROS levels across different conditions and individual patients present a significant challenge for achieving precise therapeutic outcomes. The specificity of the response to ROS is critical, as it dictates the scaffold’s ability to adapt to fluctuating biochemical signals within the injury site. One concern is the fine tuning of the scaffold material’s response to ROS levels without triggering premature or excessive responses that might lead to scaffold degradation or cytotoxicity. Additionally, the long-term stability and biocompatibility of redox-active materials, especially those involving metallic nanoparticles, remain areas requiring more extensive investigation to fully understand their interactions within the biological environment. Table 8 presents a summary of recent studies on redox and ROS-responsive scaffolds. 

#### 4.2.3. Enzyme Response

The use of enzyme-responsive materials to harness the unique catalytic and targeting capabilities of enzymes, particularly in the context of lesion tissues where enzyme levels fluctuate due to injury and inflammation, allows for the precise modulation of scaffold properties and drug release in response to specific biochemical signals within the bone defect area. The particular role of MMP-1 in degrading extracellular matrix proteins has been reported to aid in the migration of vascular endothelial cells crucial for bone healing [188]. Schoonraad et al. explored the development of a novel scaffold that enhances osteogenesis through the modification of BMP-2 with a thiol group [189]. This allowed the tethering of BMP-2 into a poly(ethylene glycol) (PEG) hydrogel, which was crosslinked with matrix metalloproteinase (MMP)-cleavable peptides [189]. These peptides respond to MMPs, enzymes that are upregulated in the bone healing process (particularly during the neovascularization phase following injury) by releasing BMP-2 precisely where it is most needed. The scaffold’s effectiveness was demonstrated through its ability to significantly elevate osteogenic markers in pre-osteoblasts, facilitated by the BMP-2 release in response to MMP activity. 

Another study by Yang et al. introduced injectable hydrogel microspheres, which are also sensitive to MMP-1 [190]. These microspheres were engineered using a microfluidic chip to encapsulate BMSC-derived exosomes (BMSC-Exos) within a matrix composed of a self-assembling peptide (KLDL-MMP1) and GelMA [6,190]. The resultant microspheres, with a uniform diameter suitable for minimally invasive injection, effectively released exosomes in response to MMP1 activity, promoting the migration and osteodifferentiation of BMSCs. In vivo tests demonstrated that these microspheres significantly aided bone repair by recruiting CD90+ stem cells through neovessels, highlighting a novel, enzyme-responsive delivery system that strategically releases therapeutic agents during critical phases of angiogenesis [190].

Further extending the scope of enzyme-responsive scaffolds, Jia et al. introduced a glucose oxidase (GOD)-responsive scaffold for diabetic patients, who often experience hindered osteogenesis due to elevated glucose levels [191]. The scaffold expands in response to increased glucose concentrations, triggering the controlled release of dexamethasone (DEX). This release mechanism simultaneously combats inflammation, promotes bone formation, and addresses the specific metabolic conditions of diabetic patients. Table 9 presents a summary of recent studies on enzyme-responsive scaffolds.

## 5. Future Perspectives and Conclusions

In this review, the potential of BTE as a viable alternative to traditional methods, such as autografts and allografts, was explored, focusing on recent advancements in biomaterials and scaffold fabrication techniques. These technologies enable scaffolds to mimic the natural bone microenvironment, enhancing osteogenesis and tissue formation. The development of smart scaffolds and bio-responsive systems capable of adapting to physical and chemical stimuli can optimize healing processes by responding dynamically to the physiological environment. Looking forward, the integration of smart nanosensors and shape memory alloys holds promise for revolutionizing BTE, offering precise control over scaffold interactions and the ability to adjust dynamically to changes within the body. We underscore the necessity for continued research into multi-responsive systems to meet the intricate demands of bone healing, emphasizing that the fusion of advanced materials and innovative fabrication techniques is crucial for advancing the efficacy of treatments in regenerative medicine and orthopedics. This ongoing integration is essential for transcending current limitations and significantly enhancing clinical outcomes in BTE.

## Figures and Tables

**Table 2:** Summary of recent studies on 3D printing techniques in scaffold fabrication.

Study Ref.	Biomaterials Used	Printing Techniques	Key Findings
[77]	PLA/β-TCP/CS with amoxicillin	Bioprinting	LbL rectangular scaffold; mechanical strength: 1.24 ± 0.53 MPa; strong antimicrobial properties, good cell viability, robust mechanical strength, and appropriate porosity were achieved
[78]	Mg–CLS/CS-coated Ti–6Al–4V	Selective laser melting	Scaffold height and diameter: 10 mm; mechanical strength: 50.3 ± 1.6 MPa; femoral bone defects in adult rabbits; surface-modified scaffold exhibited improved mechanical strength and enhanced cell adhesion, proliferation, differentiation, and the formation of new bone in a live defect model
[79]	PHBV/CaSH/CS	Fused deposition modeling	Scaffold height and diameter: 10 mm; mechanical strength: 16.6 MPa; adult male rats; increased rBMSC osteogenesis by upregulating the expression of osteogenic genes: RUNX2, COL1, OCN, OPN, and BMP2
[80]	PLGA/nHAp/CS with rhBMP2	Low-temperature deposition manufacturing	Scaffold dimension: 13 × 6 × 4 mm pore size: 431.31 ± 18.40 μm; mandibular bone defect in 13-week-old rabbits; sustained release of rhBMP2, biocompatibility in vitro, and 45.5% new bone formation were observed in vivo
[81]	GelMA scaffolds, adipose-derived stem cells	Extrusion bioprinting	Comparable cell viability of printed (79%) and non-printed (80%) scaffolds; co-cultured hydrogels show increased osteogenic differentiation with elevated levels of osteogenic markers; enhanced hydrogel calcification in pre-differentiated hADSCs
[82]	PCL, calcium magnesium phosphate	Bioprinting	Tested samples showed high biocompatibility, with over 100% live cells observed on day 3; composite scaffolds improved cell attachment and proliferation, as indicated by LDH release assays; custom-made 3D scaffolds replicated natural bone characteristics and enhanced biomineralization

**Table 3:** Summary of recent studies on temperature-responsive scaffolds.

Study Ref.	Material Composition	Method Used	Key Findings
[138]	CS, MC, VA	Sol–gel transition induced by temperature at 37 °C; magnetic stirring	VA enhances osteogenesis; ALP activity significantly increased; hydrogels are non-cytotoxic, stable, and functionally active at 37 °C; 79.65 ± 1.13% VA entrapment efficiency; swelling stable after 1 h; 69% degradation rate over 21 days; 72.5% VA released steadily over 25 days
[139]	PCL-PLLA	TIPS of PLLA after in situ polymerization of PCL-diacrylate	Can undergo deformation at a partial-melting temperature of 52 °C; retains its nanofibrous structure upon cooling to 37 °C
[140]	TMPTA, NiPAAm, AMO	cDLP	The resin composition allows adjustment of the phase transition temperature of the scaffold, enabling thermally induced mechano-stimulation of cells

**Table 4:** Summary of recent studies on mechanical- and ultrasound-responsive scaffolds.

Study Ref.	Material Composition	Method Used	Key Findings
[147]	Bi-phasic PCL/HAp	Electrospun-based thermally induced self-agglomeration	High elasticity and porosity; incorporated phenamil composite scaffolds showed less burst release and longer lasting sustained release and enhanced osteogenic differentiation of cells in vitro compared to physically surface-adsorbed phenamil
[152]	PLA embedded in SDF-1/BMP-2-loaded alginate hydrogels	3D printing and ionic crosslinking of calcium with guluronic acid chains	Pulsed ultrasound and sinusoidal continuous wave ultrasound promote the recruitment and adhesion of endogenous BMSCs to the scaffold
[153]	-	-	Significant upregulation in early osteogenic markers (RUNX2, COL1A1) and sustained increase in late markers (osteocalcin, osteopontin); mechanistic pathways involved piezo channel activation and Rho-associated protein kinase signaling
[155]	MgHAp/collagen hybrid composite	-	LIPUS stimulation 20 min/day enhanced cell viability and promoted osteogenic differentiation; improved colonization of the scaffold by human MSCs; activation of the MAPK/ERK pathway and upregulation of osteogenic and angiogenetic genes were observed—enhancing gene expression and protein release due to LIPUS stimuli

**Table 5:** Summary of recent studies on electrical-responsive scaffolds.

Study Ref.	Material Composition	Method Used	Key Findings
[162]	BT nanoparticles coated with polydopamine in polyvinylidene fluoridetrifluoroethylene (PVDF-TrFE) matrix	Surface coating; corona poling treatment	Surface potential can be adjusted up to −76.8 mV, closely aligning with the level of endogenous biopotential observed in natural bone, and it retained more than half of its original value even after 12 weeks under bone defect conditions. In vivo, it sustained the electric microenvironment, facilitating rapid bone regeneration and the formation of mature bone structures in vivo
[164]	BT/HAp composites	Hydrothermal process	d33 value: 0.61 pC/N and 0.7 pC/N for bulk ceramic composites and dry bone, respectively. Higher BT content increased the piezoelectric coefficient and dielectric constant; improved response to electrical stimuli

**Table 6:** Summary of recent studies on magnetic-responsive scaffolds.

Study Ref.	Material Composition	Method Used	Key Findings
[168]	CFO in PVDF matrix	Solvent casting, template structuring, magnetic stimulation	A 3D porous structure resembling trabecular bone, with pore sizes ranging from 5 µm to 20 µm; enhanced β-phase crystallization in PVDF, significant osteoprogenitor cell proliferation; induction of proper cellular electro-transduction processes through magnetoelectric responses of the scaffold
[169]	MBG/PCL/Fe_3_O_4_	3D printing	Magnetic heating ability was improved by the addition of Fe_3_O_4_ and did not affect the apatite mineralization ability of the scaffolds; greater expression of osteogenesis-related genes (RUNX2, OCN, BSP, BMP-2, and Col-1); ECM mineralization in human BMSCs
[170]	PCL/MNPs/placental proteins	Magnetic stimulation	Promoted osteogenic differentiation of umbilical cord MSCs

**Table 7:** Summary of recent studies on pH-responsive scaffolds.

Study Ref.	Material Composition	Method Used	Key Findings
[179]	ZIF-8 in PCL/Col membranes	Electrospinning	Responsive to acidic environments; released Zn^2+^ concentrations increased significantly under acidic conditions (pH 5.5)
[181]	CS/ZIF-8/Van	Wetspinning	70% of vancomycin released at pH 5.4 over 8 h compared to 55% at pH 7.4; increased VAN release under acidic conditions (pH 5.4) due to higher dissolution of ZIF8
[182]	PVA/ZIF-8/Van	Electrospinning	Enhanced drug release under weak acidic conditions (pH 6.5) typical of infected tissue environments

**Table 8:** Summary of recent studies on redox and ROS-responsive scaffolds.

Study Ref.	Material Composition	Method Used	Key Findings
[185]	PEM coatings/thioketal-based polymers, BMP-2	LbL	Thioketal-based polymers specifically cleaved by physiologic doses of ROS, unlike typical non-specific hydrolysis; enhanced ROS-mediated protein delivery in vitro. A 50% increase in bone regeneration over less sensitive formulations; nearly a threefold extension in BMP-2 delivery half-life compared to conventional hydrolytically sensitive coatings
[186]	PGLA/nano-HAp	LbL, surface coating	A responsive H_2_O_2_ detection system via the addition of silk fibroin/horse peroxidase; targeted human BMSCs with uptake within 2 h; exocytosis occurring 6 h after cellular uptake; can deliver antioxidants directly to sites of I/R injury and enhance the survival and functionality of hBMSCs in oxidative environments
[187]	MBG/Co^2+^	-	Co^2+^ acts as a chemical inducer of HIF-1α, simulating a hypoxic environment that enhances angiogenic and osteogenic responses; enhanced VEGF protein secretion, indicating improved angiogenesis; supported the attachment and proliferation of BMSCs

**Table 9:** Summary of recent studies on enzyme-responsive scaffolds.

Study Ref.	Material Composition	Method Used	Key Findings
[189]	PEG/MMP-cleavable peptide/BMP-2	Crosslinking, thiolation of BMP-2, thiol-norbornene click chemistry	Significant increase in expression levels of osteogenic markers Bglap and Ibsp in the presence of tethered BMP-2 and enhanced cell differentiation; confirmed the activation of the BMP canonical signaling pathway (via SMAD 1/5/8 route), which is critical for osteogenesis
[190]	GelMA/MMP-1/KLDL-MMP1/BMSC-Exos	Microfluidic chip	Responsive to MMP1 and enabling targeted and controlled release of exosomes; enhanced bone repair in vivo by recruitment of CD90+ stem cells through neovessels
[191]	PCL/CS/DEX/GOD	Electrospinning, genipin	Scaffolds effectively promoted osteogenic differentiation of MC3T3-E1 cells in high-glucose conditions

## Data Availability

No new data were created or analyzed in this study. Data sharing is not applicable to this article.
